# Interaction between the level of immunoglobulins and number of somatic cells as a factor shaping the immunomodulating properties of colostrum

**DOI:** 10.1038/s41598-021-95283-1

**Published:** 2021-08-03

**Authors:** Kamila Puppel, Marcin Gołębiewski, Jan Slósarz, Grzegorz Grodkowski, Paweł Solarczyk, Piotr Kostusiak, Kinga Grodkowska, Marek Balcerak, Tomasz Sakowski

**Affiliations:** 1 Animal Breeding Department, Institute of Animal Science, Warsaw University of Life Sciences, Ciszewskiego 8, 02-786 Warsaw, Poland; 2 Institute of Genetics and Animal Biotechnology, Polish Academy of Science, Jastrzębiec, Postępu 36A, 05-552 Magdalenka, Poland

**Keywords:** Zoology, Immunology, Inflammation

## Abstract

The aim of this study was to investigate the association between immunoglobulins and SCC as a factor in shaping the content of the immunostimulatory components of colostrum. Seventy-eight multiparous Polish Holstein–Friesian cows were selected for the experiment. Colostrum samples were collected immediately after calving (up to a max. of 2 h). The cows were divided into groups according to the following levels: Immunoglobulins (IG class)—(IG_1_) over 50 g/L, (IG_2_) up to 50 g/L; SCC class—(SCC_1_) up to 400 000/ml, (SCC_2_) 400–800 000/ml, (SCC_3_) over 800 000/ml. Colostrum assigned to the IG_1_ SCC_1_ group had a statistically significant higher (p ≤ 0.01) concentration of both whey proteins and fatty acids compared to the IG_1_ SCC_2_ and SCC_3_ groups. The concentration of IgG, IgM, and IgA was shown to be higher in IG_1_ SCC_1_ than IG_2_ SCC_3_ by 226%, 149%, and 115%, respectively. The concentration of lactoferrin was shown to be higher in IG_1_ SCC_1_ than IG_2_ SCC_3_ by 149%. The determination of colostrum quality based on the concentration of immunoglobulins in the colostrum may not be sufficient because serum IgG concentrations at birth show a linear increase relative to colostrum SCC. A breakdown of colostrum into quality classes, taking into account the level of SCC, should therefore be introduced.

## Introduction

The concentration of colostrum immunoglobulins (Ig) varies with the cow’s health status^1^, amount of colostrum produced, calving season^2^, breed, age^3,4^, production system^5^, length of non-lactating period^6^, prepartum milking^7^, time delay between parturition and first milking^8,9^, and heat stress^10^. Additionally, increased levels of Ig were demonstrated in cows inoculated with vaccines containing *E. coli*, *Coronavirus* and *Rota* antigens^11^. In cow's milk, IgG is the main isotype, followed by IgA and IgM^12^. IgG plays a role in the immune response to infections, while IgA is involved in protecting the mucous membranes, and IgM is the first line of defense against infections^13^. Due to its structure in ruminants, the placenta prevents the transfer of antibodies to the fetus, therefore the concentration of Ig in the blood serum of calves is low^14^. The neonate is immunonaive and dependent on passively acquired maternal immunoglobulins^15^. Likewise, colostrum intake by calves is crucial to protect against gastrointestinal tract infections. This uptake of IgG occurs partly via passive transport across the epithelium when it is not yet fully closed, but also actively via the FcRn, the neonatal IgG receptor that is expressed on the intestinal epithelium in neonatal calves^16^. The functional but naive immune system of the newborn does not permit an effective immune response for the first three weeks of life^17^. A very important aspect of calf rearing is the feeding of high-quality colostrum, in conjunction with appropriate frequency, quantity, and temperature^19^. When one of these elements does not meet the standards, the consequence is the calves’ increased susceptibility to diarrhea and infections, which in turn has a direct impact on production economics. McGuirk and Collins^18^ reported that calves with failed passive transfer have mortality and morbidity risks up to six times higher than those that have succeeded.

Colostrum is also one of the factors, that is responsible for the microbial colonization of the gastrointestinal tract. Puppel et al.^19^ reported that high-quality colostrum stimulated significant development of *Lactobacilli* and *Bifidobacterium* spp., simultaneously reducing *Coliforms* and *Enterococci*. Strain growth depends on lactoferrin (LF) and lysozyme (LZ) concentration, which are involved in maintaining the balance of the intestinal microflora, due to their properties^20–22^. Additionally, lactoferrin and casein inhibit lipid peroxidation as well as the formation of peroxide radicals and iron oxide^23^.

The quality and quantity of cows’ mammary gland secretions are closely related to udder health. Puppel et al.^1^ reported that in colostrum from the first milking, the IgG concentration was two-fold greater and the C18:2 cis9trans11 three-fold greater in colostrum with somatic cell count (SCC) ≤ 400 000 cells/ml, than in colostrum with ≥ 400 000 cells/ml. It should be emphasized that fatty acids, are antibacterial agents that destabilize bacterial cell membranes^24^, due to their amphipathic properties. The consequence is increased membrane permeability and cell lysis, as well as inhibition of the enzymatic activity of the membrane^25^. Calves cannot fight infectious agents because their immune system is underdeveloped at the time of birth. After birth, they therefore depend immunologically on the successful, passive transfer of maternal Ig via colostrum^26^. Ferdowsi Nia et al.^27^ reported that feeding neonates with high SCC colostrum decreased serum IgG levels and also increased the incidence of diarrhea in calves.

The aim of this study was to investigate the association between immunoglobulins and SCC as a factor in shaping the content of the immunostimulatory components of colostrum in the first milking after calving.

## Results

**Figure 1:**
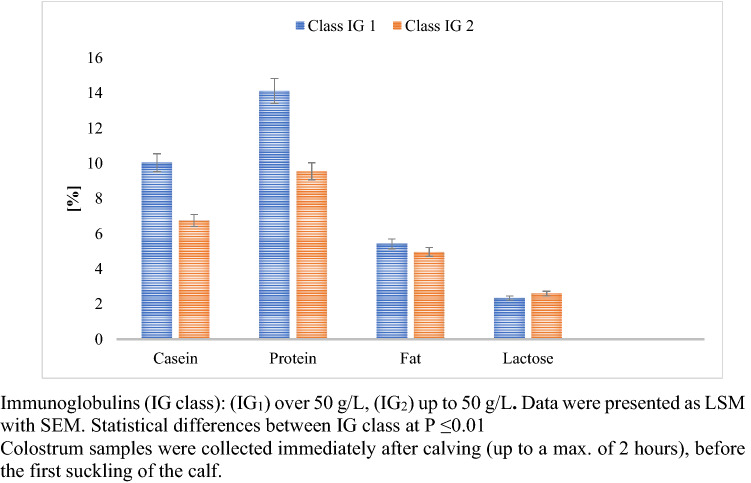
Changes in colostrum’s gross composition for different Ig concentrations.

Colostrum samples were collected from multiparous cows and evaluated for IgG and SCC content. Based on IgG and SCC samples were assigned to the following categories: the level of: Immunoglobulins (IG_1_, IG_2_) and Somatic Cell Count class (SCC_1_, SCC_2_, SCC_3_). The colostrum assigned to IG_1_ was characterized by significantly higher concentrations of casein, total protein, and fat. The study showed that IG class had a statistically significant (p ≤ 0.01) effect on the formation of the level of colostrum’s functional parameters in the first milking after calving (Fig. 1).

**Figure 2:**
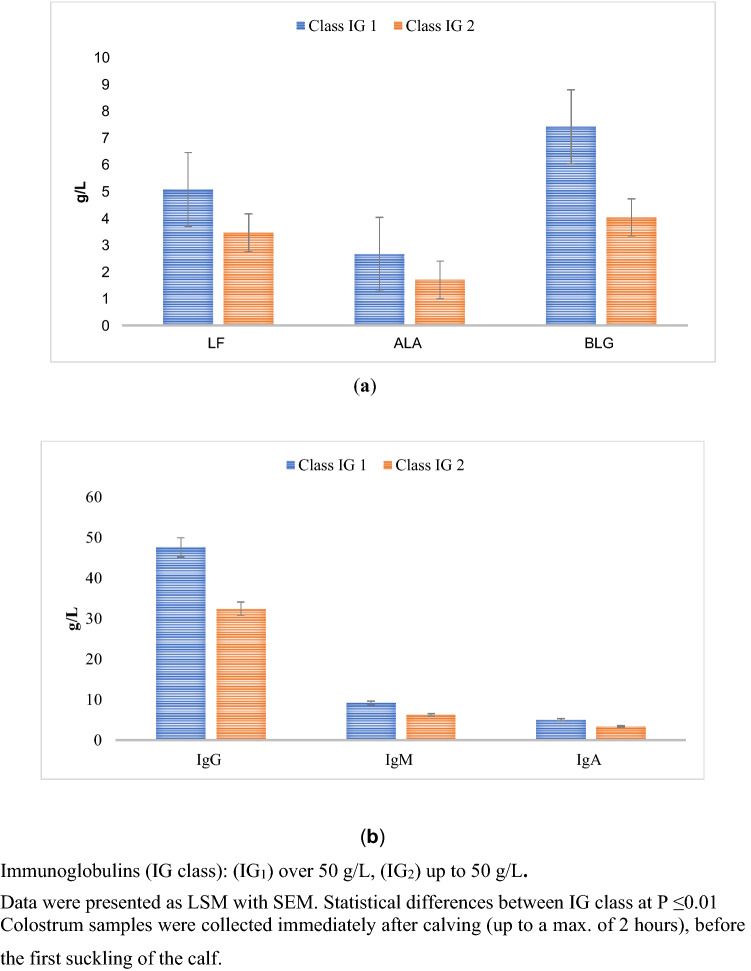
Changes in whey protein content (**a**) LF, ALA, BLG, (**b**) IgG, IgM, IgA for different IG concentrations.

In comparing IG_1_ and IG_2_, the concentration of lactoferrin (LF), α-lactalbumin (ALA), and β-lactoglobulin (BLG) was higher by almost 30% for IG_1_ than for IG_2_ (Fig. 2a), while the G, M, and A immunoglobulins were higher by almost 35% (Fig. 2b). Based on the analysis of the obtained results, a statistically significant (p ≤ 0.01) relationship between Ig class and the level of bioactive whey proteins was demonstrated (Fig. 2a,b).

**Figure 3:**
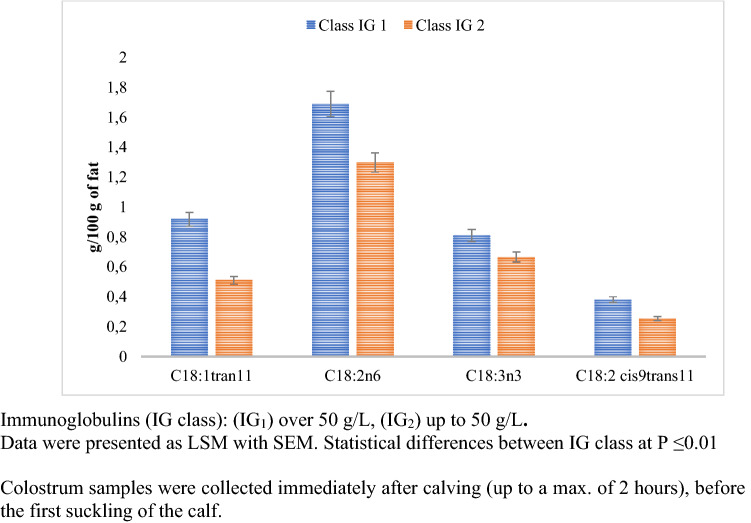
Changes in selected fatty acids’ content for different IG concentrations.

Colostrum assigned to the IG_1_ group had significantly higher concentrations of C18:1 trans11, C18:2 n-6, C18:3 n-3, and C18:2 cis9 trans11. The study showed that the IG class had a statistically significant (p ≤ 0.01) effect on the formation of bioactive fatty acids during the first milking after calving (Fig. 3).

**Figure 4:**
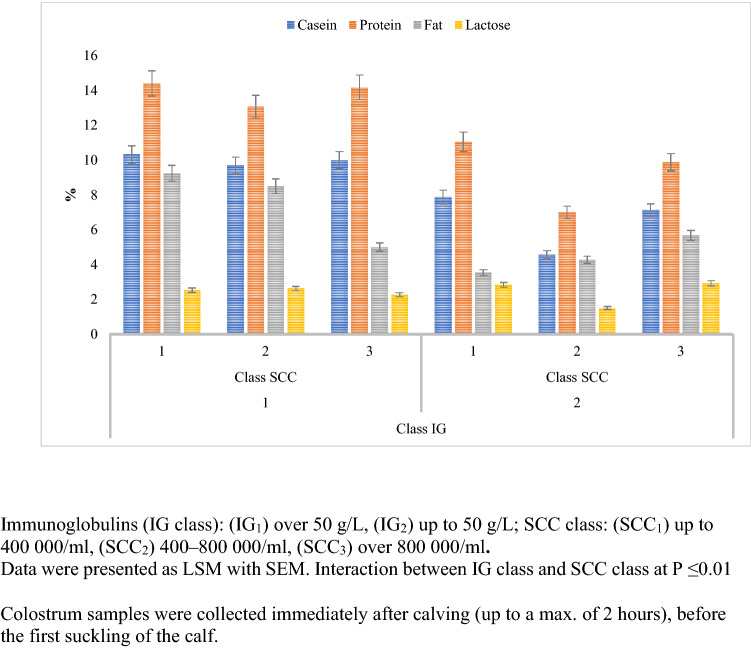
Interaction between the level of immunoglobulins and SCC as a factor shaping the basic chemical composition of colostrum [%].

The colostrum assigned to the IG_1_ and SCC_1_ groups had significantly higher concentrations of protein, fat, and casein, compared to the IG_1_ SCC_2_ and SCC_3_ groups (Fig. 4). The study showed that the Ig × SCC interaction had a statistically significant (p ≤ 0. 01) effect on the formation of the functional parameters of colostrum during the first milking after calving.

**Figure 5:**
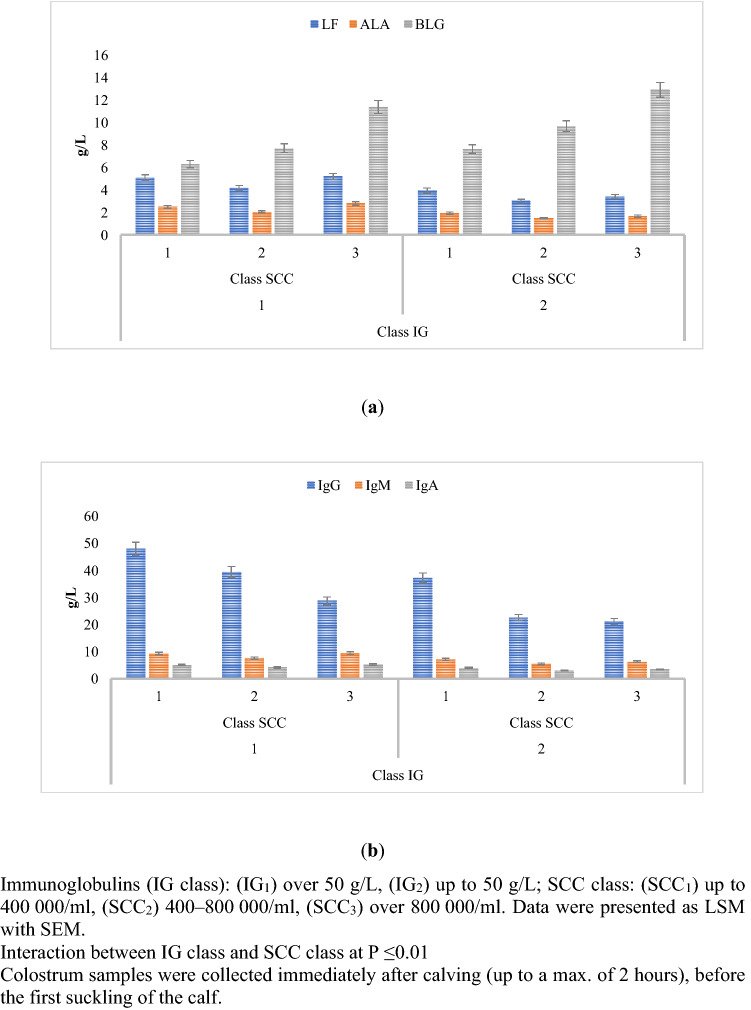
Interaction between the level of immunoglobulins and SCC as a factor influencing the level of whey proteins (**a**) LF, ALA, BLG, (**b**) IgG, IgM, IgA.

Colostrum assigned to the IG_1_ SCC_1_ group had a statistically significant (p ≤ 0.01) higher levels of bioactive whey proteins compared to the IG_1_ SCC_2_ and SCC_3_ groups (Fig. 5a,b). The study showed that the Ig x SCC interaction had a statistically significant (p ≤ 0.01) effect on the level of lactoferrin, α-lactalbumin (Fig. 5a), and immunoglobulin during the first collection after calving (Fig. 5b).

The lowest level of β-lactoglobulin in IG_1_ SCC_1_ was reported as 6.311 g/L, while the highest level in IG_2_ SCC_3_ was 12.941 g/L. The study showed that the Ig x SCC interaction had a statistically significant (p ≤ 0.01) effect on the formation of β-lactoglobulin (Fig. 5a).

**Figure 6:**
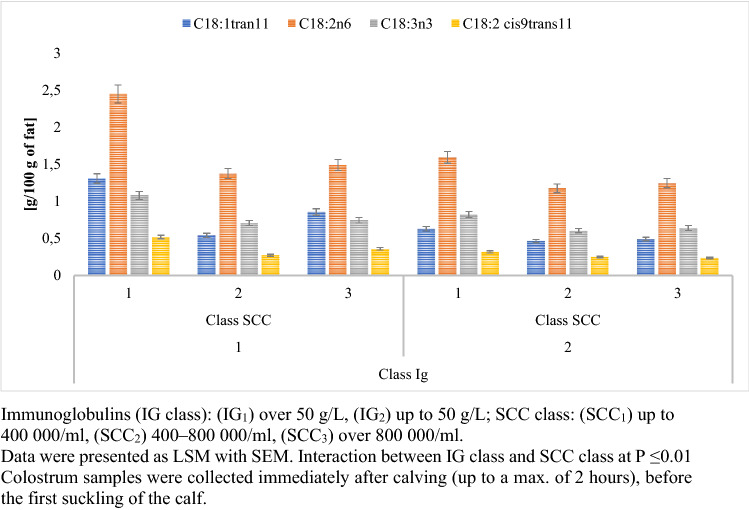
Interaction between the level of immunoglobulins and SCC as a factor influencing the level of selected fatty acids.

The colostrum assigned to the IG_1_ SCC_1_ group had a statistically significant (p ≤ 0.01) higher concentration of bioactive fatty acids compared to the IG_1_ SCC_2_ and SCC_3_ groups (Fig. 6). The highest level of C18:2 cis9trans11 in IG_1_ SCC_1_ was demonstrated to be 0.515 g/100 g fat, while the lowest level in IG_2_ SCC_2_ was 0.231 g/100 g fat. The study showed that the Ig x SCC interaction had a statistically significant (p ≤ 0.01) effect on the formation of Conjugated Linoleic acid (CLA) (Fig. 6).

## Discussion

The most important immunostimulating components in colostrum are immunoglobulins, which affect the immunity of the calf organism^28–30^. In order for the body to take full advantage of these components, attention is paid to factors such as the quality of the colostrum, the time elapsed since birth (which is crucial, due to the decreasing absorption capacity of the substance through the intestinal epithelium), and the quantity of colostrum^2,31^. IgG are powerful effector molecules that can mediate tissue inflammation by complement activation, and by engaging classical FcγRs and C-type lectin receptors^32^. The concentration of IgG, IgM, and IgA was shown to be higher by 226%, 149%, and 115%, respectively, in IG_1_ SCC_1_ than in IG_2_ SCC_3_ (Fig. 5b). Maunsell et al.^6^ reported that altered cell function caused by mastitis might reduce IgG_1_ transport, and result in low colostral IgG_1_ concentrations in infected glands. Bollinger et al.^33^, reported, that intestinal epithelium has the capability of transporting Ig into the lumen via the expression of the polymeric immunoglobulin receptor. However, bacteria can bind free IgG in the intestine, or block IgG molecules from being taken up and transported into enterocytes, thereby impairing IgG absorption^34^. It is assumed that good quality colostrum is characterized by a high concentration of IgG. Concentrations of this component should be higher than 50 g/L^35^. The calf’s intestinal absorption phase is nonspecific for the class of immunoglobulins and is operative, essentially, only during the first 24 h after birth^36^. With the average absorption of immunoglobulins through the intestine (20–30%), during the first six hours of life, the calf should ingest 100–200 g of Ig. This allows adequate passive transfer, which is guaranteed by the colostrum assigned to IG_1_ SCC_1_. Therefore, it can be concluded that colostrum characterized by a higher concentration of IG has higher immunostimulatory properties.

Immunomodulating and/or antimicrobial substances, including lactoferrin and lysozyme, are present in mammary secretions and may contribute to the protection of neonates^37^. The study showed that the Ig x SCC interaction had a statistically significant (p ≤ 0.01) effect on lactoferrin concentration. The concentration of LF was shown to be higher in IG_1_ SCC_1_ than in IG_2_ SCC_3_ by 149% (Fig. 5a). In juveniles there is an initial lack of intestinal barrier, so LF has the opportunity to stay in the intestine longer and consequently penetrate the bloodstream^38^. LF increases both in vivo and in vitro enterocyte differentiation. In addition, LF was found to increase in vitro enterocyte proliferation resulting in higher cell density in cell flasks^39^. Therefore, it can be concluded that colostrum characterized by a higher concentration of lactoferrin has higher immunostimulatory properties.

Bravo-Santano et al.^40^ and Kenny et al.^41^ reported that the innate immune system in epithelial cells takes advantage of long chain free fatty acids such as C18:2 n-6 and C18:1 cis9, because they are considered bactericidal or bacteriostatic depending on their structural characteristics^25^. The concentration of C18:1 trans11, C18:2 n-6, C18:3 n-3, and C18:2 cis9trans11 was shown to be higher in IG_1_ SCC_1_ than in IG_2_ SCC_3_ by 267%, 197%, 169%, and 222%, respectively (Fig. 6). Studies have shown that the de novo synthesis of fatty acids and the acylation of long-chain fatty acids using glycerol can be impaired in mammary glands during the inflammatory process. Meydani et al.^42^ reported that n-3 FAs decrease bacterial lipopolysaccharide-induced production of the pro-inflammatory cytokines IL-1 and TNF from peripheral blood lymphomonocytes. However, in the event of inflammation, the membrane’s enrichment of n-3 FAs reduces the ability of the endothelial cells to respond to stimulation by bacterial lipopolysaccharide, IL-I, IL-4, or TNF in terms of the intercellular adhesion molecule-1, as well as soluble mediators, such as IL-6 and IL-8, that are able to provide positive feed-back to amplify the inflammatory response^43,44^. Thus, it can be concluded that colostrum of good cytological quality significantly influences the natural defensive mechanisms of calves, because the above-mentioned acids show anti-inflammatory effects.

In conclusion, the level of immunostimulatory components in colostrum is variable, and one of the modulating factors is cytological quality. A breakdown of colostrum into quality classes, taking into account the level of SCC: up to 400 000/ml, 400–800 000/ml, over 800 000/ml., should therefore be introduced.

## Methods

All cows were handled in accordance with the regulations of the Polish Council on Animal Care; and the Second Ethics Committee for Animal Experimentation in Warsaw of the Ministry of Science and Higher Education (Poland) reviewed and approved all procedures (Approval number: WAWA2/086/2018). During the experiment, the cows were under veterinary care. Dry cows were fed according to the guideline’s rules of the Nutrient Requirements Committee.

Seventy-eight multiparous (in second lactation) Polish Holstein–Friesian cows were selected for the experiment. Colostrum samples (250 ml) were collected in sterile plastic containers containing the preservative Mlekostat CC immediately after calving (up to a max. of 2 h), before the first suckling of the calf, and then transported to the Warsaw University of Life Sciences and stored frozen (− 20 °C) until the planned analysis.

After a preliminary analysis of the samples, cows were divided into groups according to the level of:

1. Immunoglobulins (IG class):
  IG_1_ over 50 g/L (range of IG values: 60–78 g/L; n = 27),
  IG_2_ up to 50 g/L (range of IG values: 32–50 g/L; n = 51)
2. Somatic Cell Count class (SCC class):
  SCC_1_ up to 400 000/ml, range of SCC values: 150 000–400 000 cells/ml; n = 27
  SCC_2_ 400–800 000/ml, n = 28,
  SCC_3_ over 800 000/ml (range of SCC values: 820 000–1500 000 cells/ml; n = 23).

### Chemical analysis

The basic chemical composition of colostrum (fat, protein, lactose, casein, density) was determined using a Milko-Scan FT-120 analyzer (Foss Electric, Denmark).

Cytological quality (somatic cell count: SCC) was established using a Somacount 150 analyzer (Bentley, Warsaw, Poland).

Each sample of colostrum was centrifuged for 15 min at 5,000 × *g* in a microcentrifuge, and then the fat layer was removed. The remaining solubilized sample (5 mL) was heated to 40 °C and then, 10% solution of acetic acid was added to precipitate the casein fraction. After thawing, each sample was centrifuged for 15 min at 14,000 × *g* in a microcentrifuge. The supernatant was filtered through a nylon filter and used in further steps of the analysis: whey proteins and immunoglobulins.

Concentrations of whey proteins were determined using an Agilent 1100 Series RP-HPLC (Agilent Technologies, Waldbronn, Germany). Separations were performed at ambient temperature using solvent gradient on a Jupiter column C18 300A (Phenomenex, Torrance, CA, USA). The chromatographic conditions were as follows. Solvent A was acetonitrile (Merck, Darmstadt, Germany), water (Sigma-Aldrich) and trifluoroacetic acid (Sigma-Aldrich) in a ratio of 70:930:1 (v/v/v). Solvent B was acetonitrile, water, and trifluoroacetic acid in a ratio of 930:70:1(v/v/v). The flow rate was 1.4 ml/min and the detection wavelength was 220 nm. All samples were analyzed in duplicate. The identification of peaks as lactoferrin and lysozyme was confirmed by comparing them with the standards (Sigma-Aldrich, USA).

Concentrations of immunoglobulins (G, M, A) were determined using an Agilent 1100 Series RP-HPLC (Agilent Technologies, Waldbronn, Germany). The chromatographic conditions were as follows. Solvent A was acetonitrile (Merck, Darmstadt, Germany), water (Sigma-Aldrich) and trifluoroacetic acid (Sigma-Aldrich) in a ratio of 20:980:1 (v/v/v). Solvent B was acetonitrile, water, and trifluoroacetic acid in a ratio of 980:20:1(v/v/v). The column was first equilibrated at 25% mobile phase A for 2 min at a 2 mL/min flow rate. The elution was performed as a gradient of mobile phase A, from 25 to 60% over 5 min at 2 mL/min. The detection wavelength was 280 nm. All samples were analyzed in duplicate. The identification of peaks as immunoglobulins was confirmed by comparing them with the standards of Bovine Ig (Sigma-Aldrich, USA).

Fatty acid methylation was carried out using the *trans-*esterification method PN-EN ISO 5509:2000^45^. Concentrations of fatty acids were determined using an Agilent 7890 GC gas chromatograph (Agilent Technologies, Waldbronn, Germany) and Varian Select FAME column. The separation was performed at pre-programmed temperature: 130 °C for 1 min; 130–170 °C at 6.5 °C min − 1; 170–215 °C at 2.75 °C min^−1^; 215 °C for 12 min, 215–230 °C at 20 °C min^−1^ and 230 °C for 3 min. Helium at a flow rate of 25 cm s^−1^ and constant pressure was used as the carrier gas, the injector temperature was 240^◦^C, and the detector temperature was 300 °C. All samples were analyzed in duplicate. Each peak was identified using pure methyl ester standards (Supelco, USA).

### Statistical analysis

The data were compiled statistically by a multi-factor analysis of variance using the least squares method. The decomposition of bioactive components was checked using the Shapiro–Wilk test. All tests were conducted using an IBM SPSS 23^46^. After a preliminary analysis of the samples, cows were divided into groups according to the level of Immunoglobulins (IG class): (IG_1_) over 50 g/L, (IG_2_) up to 50 g/L; SCC class: (SCC_1_) up to 400 000/ml, (SCC_2_) 400–800 000/ml, (SCC_3_) over 800 000/ml.

The statistical model was:

$$ {\text{Y}}_{{{\text{ijk}}}}  = \upmu  + {\text{A}}_{{\text{i}}}  + {\text{B}}_{{\text{j}}}  + ({\text{A}}_{{\text{i}}}  \times {\text{B}}_{{\text{j}}} ) + {\text{e}}_{{{\text{ijk}}}}  $$

where: y is the dependent variable, µ is the overall mean, A_i_ is the fixed effect of the IG class (I = 1 − 2), B_j_ is the fixed effect of the SCC class, A_i _× B_j_ is the interaction between IG class and SCC class, and e_ijk_ is the residual error.

### Ethics approval

The Second Ethics Committee for Animal Experimentation in Warsaw of the Ministry of Science and Higher Education (Poland) reviewed and approved all procedures (Approval number: WAWA2/086/2018). All cows were handled in accordance with the regulations of the Polish Council on Animal Care, and the Warsaw University of Life Sciences Care Committee reviewed and approved the experiment and all procedures carried out in the study.

## Data Availability

All data generated or analyzed during this study are included in this published article. The datasets used and/or analyzed in the current study are available from the corresponding author on reasonable request.
